# Primary extradural meningioma with a history of traumatic head injury during infancy: A case report

**DOI:** 10.1016/j.ijscr.2024.109743

**Published:** 2024-05-08

**Authors:** Elliot J.D. Crene, Claudia J.K. Cockburn, William J. Cockburn

**Affiliations:** a Neurosurgical Department, Gold Coast University Hospital, 1 Hospital Bld, Southport 4215, Queensland, Australia; b Intensive Care Unit, Gold Coast University Hospital, Queensland 4215, Australia; c The Welsey Hospital, Brisbane, Queensland 4066, Australia

**Keywords:** Neurosurgery, Oncology, Meningioma, Rare, Surgery, Scalp, Trauma

## Abstract

**Introduction and importance:**

Meningiomas are an extra-axial tumour arising from arachnoid cells and are typically benign and slow growing. Primary extradural meningiomas refer to meningiomas that arise outside the subdural compartment and are extremely rare (0.3 % of meningiomas).

**Case presentation:**

A 42-year-old female presented to her primary health care provider with a 2-year history of a painful mass on her left forehead with a past medical history of a traumatic brain injury and intracranial hematoma from a motor vehicle accident when she was 11 months old. An ultrasound reported as likely sebaceous cyst. The lesion was resected and sent for pathological examination. The diagnostic summary reported an ectopic subgaleal left frontal meningioma WHO Grade 1.

**Clinical discussion:**

Extracranial meningiomas have been divided into two classifications; primary extracranial meningiomas and secondary extracranial meningiomas. In the female population group 88 % of extracranial meningiomas found on the scalp/skin are grade 1 meningiomas. Most extracranial meningiomas are diagnosed after histology examination, due to the rarity. They can arise via entrapment of arachnoid cells during embryologic development and from traumatic events displacing arachnoid cells.

**Conclusion:**

The authors suggest that the patient's aetiology of her PEM is from the entrapment of arachnoid islet cells secondary to her traumatic brain injury during infancy. Interestingly, the patients' symptoms began 40 years post trauma. Other case studies of this rare tumour have correlated a shorter time period between the trauma and the diagnosis. We suggest that all patients should have radiographic and histologic investigations of scalp masses.

## Highlights

• Based on the postulated mechanisms on how extradural meningiomas arise, the authors suggest that the patient's aetiology of her PEM is from the entrapment of arachnoid islet cells secondary to her traumatic brain injury during infancy.
• Interestingly the patients' symptoms began 40 years post trauma. Other case studies of this rare tumour have correlated a shorter time period between the trauma and the diagnosis.
• We suggest that all patients should have radiographic investigations of scalp masses that have history of head trauma.

## 1 Introduction and importance

Meningiomas are an extra-axial tumour arising from arachnoid cells and are typically benign and slow growing [1]. The location of meningiomas arise anywhere that arachnoid cells are found which typically include between brain and skull, along the spinal cord and/or within the ventricles. Primary extradural meningiomas (PEM) refer to meningiomas that arise outside the subdural compartment and are extremely rare, accounting for only 0.3 % of meningiomas [1,2]. Meningiomas have been reported to be linked to head trauma, with majority of cases reported to be intradural in location. To date, there are only several case reports with PEM related to trauma. We present a rare case of a PEM that presented over 40 years post traumatic head injury confirmed by histopathology [12,16].

This case report has been reported in line with the SCARE criteria and PROCESS guidelines [14,15].

## 2 Case presentation

A 42-year-old female presented to her primary health care provider with a 2-year history of a painful mass on her left forehead with a past medical history of a traumatic brain injury and intracranial hematoma from a motor vehicle accident when she was 11 months old. She has a CT head with contrast showing evidence of gliosis in the right basal ganglia suggest previous trauma (Fig. 2). She had left forehead mass over the supraorbital skin that had been increasing in size over several years and was mildly tender on palpation. No skin changes were noted on examination. She has residual left sided hemiparesis from the accident. She has no history of any neurosurgical operations. She has no other co-morbidities and no regular medication. She has a family history of sebaceous cysts only without any history of known brain lesions/meningiomas.

**Fig. 1:**
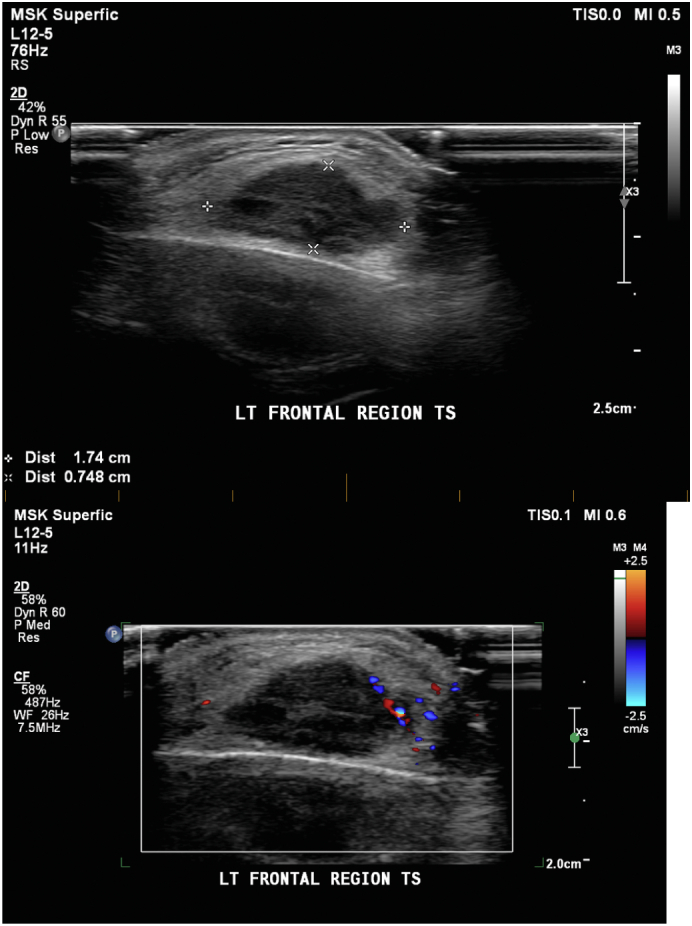
Ultrasound images of the patient's ECM pre operatively showing a hypoechoic lesion measuring 17x7x21mm, with no significant vascularity.

An ultrasound examination was organized by her primary health care doctor which was reported as likely sebaceous cyst (Fig. 1) with features of a hypoechoic lesion 17x7x21mm in size, with no increased vascularity or bone involvement. She was referred to a plastics and reconstructive surgeon specialist for further management. The lesion was resected and sent for pathological examination. Intraoperative findings included that the lesion was noted to have an intimate association with the left frontal bone within the subgaleal plane. The surgery was performed by a senior plastic surgeon, Dr. W Cockburn (Fig. 3).

**Fig. 2:**
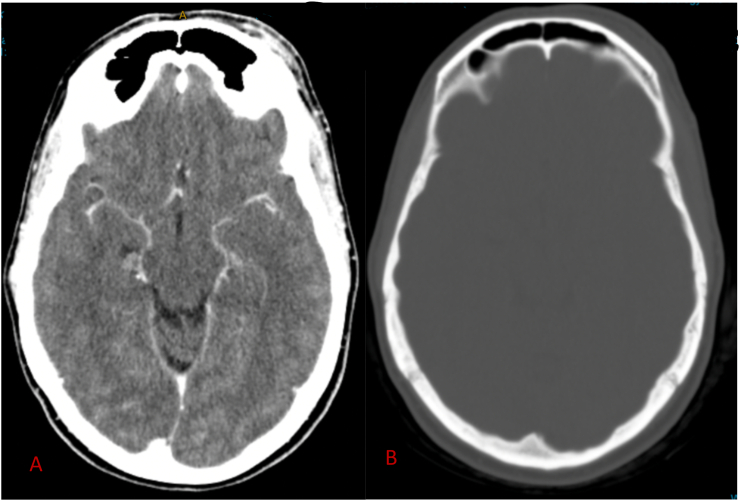
(A and B): A Computer Tomography (CT) scan with pre and post contrast that was performed 10 days post-surgery. Fig. 2A is an axial CT bone window at the level on the frontal sinuses showing no osseous involvement or destruction. Fig. 2B shows post contrast enhancement at the left supraorbital subcutaneous area, which could either be secondary to post-surgical changes or residual PEM. There is no pre operative CT or MRI to compare to.

Macroscopically, the lesion was reported as an irregular shaped mass of grey yellow fibrofatty tissue *24x19x10mm.* Microscopically the lesion was a cellular tumour which comprised of ovoid cells arranged in nodules with whorled pattern containing single nucleolus only and some mitotic activity (Fig. 2). On further immunohistochemistry, the tumour cells were positive for EMA, cytokeratin AE 1/AE3 and PR. There was also focal S-100 and variable CD99 staining. The Ki-67 proliferation index was low (less than 5 % with a focal area amounting to 20 %). The diagnostic summary reported an ectopic subgaleal left frontal meningioma WHO Grade 1.

**Fig. 3:**
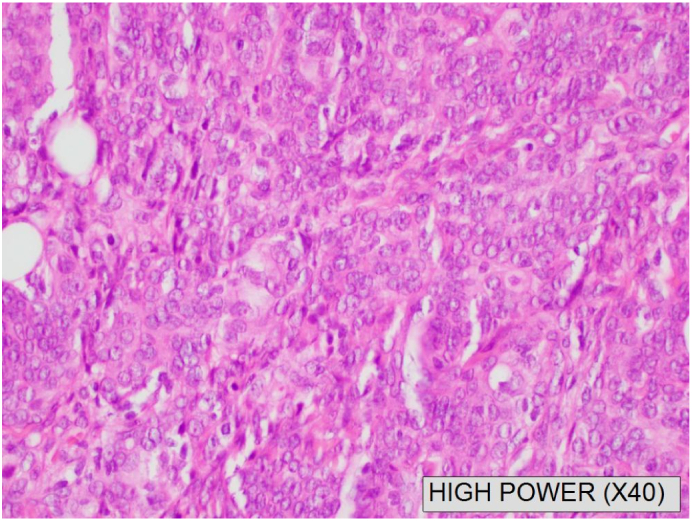
A high-powered haematoxylin and eosin (H + E) stained slide of the patients excised scalp mass showing ovoid cells in a whorled pattered, consistent with a meningioma.

**Fig. 4:**
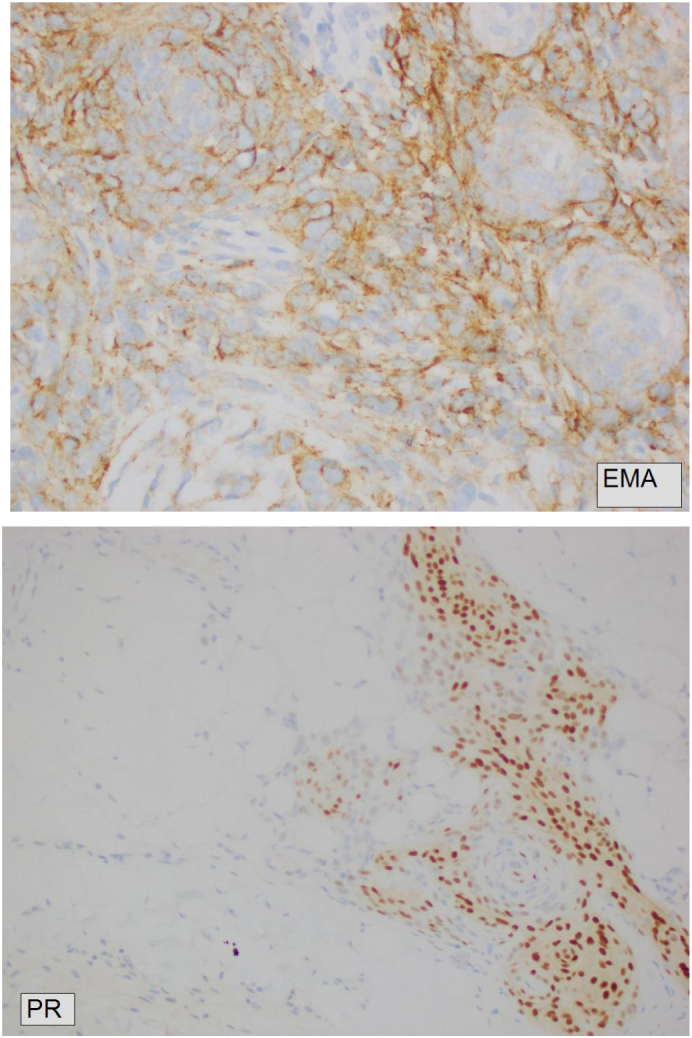
A slide of the patient's scalp mass staining positive for both epithelia membrane antigen (EMA) and progesterone receptor (PR).

Post operatively, a CT brain and neck with pre and post contrast was organized to look for intracranial pathology. There was no broad based dural enhancing lesion to suggest an intracranial meningioma. No destructive osseous lesion or cervical adenopathy was identified. Clinically the patient had an uncomplicated recovery and to date has no clinical reoccurrence of her PEM at a 1 year follow up.

## 3 Clinical discussion

Meningiomas constitute 15 % of all intra-cranial tumours and are the most common benign brain tumour [3]. Extra-cranial meningiomas are rare, making up less than 2 % of all meningiomas [4].

Following a common theme for most brain tumours, there has been no established definite aetiological factor for meningiomas [3]. Epidemiological studies have shown correlation between radiation exposure and incidence of meningiomas, such as the Nagasaki atomic bomb patients [5]. Case studies have also identified head trauma as a possible predisposing factor for the development of meningiomas, however epidemiological studies do not support this [6]. Meningiomas usually arise a single tumour, however patients with NF2 can develop multiple meningiomas, albeit intracranial. In a case series of analysis of extra-cranial meningiomas, no patients had syndrome associated meningiomas [7].

Extracranial meningiomas have been divided into two classifications; primary extracranial meningiomas and secondary extracranial meningiomas. Primary ECM are entirely extracranial in origin, whereas secondary ECM are intradural meningiomas with extracranial extension [7]. Meningiomas arise from the arachnoid cap cells in the arachnoid meninges layer [3], which embryologically are derived from neural crest cells [7].Rushing et al., analysed 146 case studies of extra-cranial meningiomas, with 74 cases being female and 72 male. Patient's age ranged from 3 months to 88 years old with a mean presentation 42.4 years, which is very similar to the author's patients age at presentation (40 years old). The mean duration of symptoms was 27.1 months, with a range of 2 weeks to 240 months. In the female population group, they found that the most common site for extra cranial meningiomas was the ear and temporal bone (22 %) and the second most common was the scalp 22% [7].88% of extracranial meningiomas found on the scalp/skin are grade 1 meningiomas. Only 5 % are anaplastic/grade 3 [7].

There are four different mechanisms postulated on how extra-cranial meningiomas arise. Through a pathological mechanism, arachnoid cells emerge outside the meningeal neurological axis and subsequently develop into extra cranial meningiomas. This may occur due to: extra neuraxial extension of an intracranial meningiomas; entrapment of arachnoid cells during embryologic development; traumatic events displacing arachnoid cells; or, multipotential mesenchymal cells differentiating to form extra-cranial meningiomas [7]. Some authors have hypothesised that an external stimulus such as trauma can induce neoplastic changes in arachnoid cap cells that entered the calvarium during the embryonic period as mechanism for trauma and PEMs^12.^

Most extracranial meningiomas are diagnosed after histology examination, due to the rarity of these tumours [8]. There is also commonly a lack of clear a radiographic finding due to the extra cranial location and lack of dural attachment makes diagnosis difficult [8]. Literature suggests that CT with contrast and MRI imaging are the superior modalities to assess anatomical relationships EDM. Contrast CT studies usually show a hyperdense lesion that enhances with contrast [9]. Many EDMs are also osteoblastic. MR allows assessment of soft tissue extension and vascularization [8]. On ultrasound imaging, meningiomas are usually hyperechoic with a homogenous pattern. Calcifications are also be seen in the lesion [10].

The patient in our case study had a pre operative ultra-sound showed a hypoechoic lesion with no increased vascularity (Fig. 1). A post-operative CT head with contrast and subsequent MRI with gadolium which did not show any intradural pathology, thus confirming a primary extracranial meningioma (Fig. 2).

Microscopically, ECM tumours are divided histologically into WHO grades I to III, an identical grading system to that of intracranial meningiomas [7]. Immunohistochemistry profiles of ECMs all express epithelia membrane antigen (EMA) and vimentin [7]. S100 staining and progesterone receptor positivity can also be seen [7]. The Ki-67 index is a marker for cell proliferation in meningiomas [11]. For our patient, all tumour cells were positive for EMA and had focal staining for S100 with a Ki-67 index less than 5 %. The overall pathological diagnosis was a WHO Grad 1 meningioma, however the lesion involved peripheral edges (Fig. 4).

In review of multiple case studies and reviews, surgical excision remains to gold standard of treatment with adequate margins of normal surround tissue if feasible [8]. Radiotherapy adjuvant treatment in partially resected primary extradural meningiomas is recommended. In WHO Grade I PEM, chemotherapy is not recommended [9]. In a literature review by Mattox et al., they suggested yearly MRI follow up for 5 years for low grade PEMs that were completely resected [9]. The prognosis of extra-cranial meningiomas tends to be favourable, with an overall median survival time of 28 years [7].

Prior to our report, only 14 cases studies have been described in patients with a PEM and a history of head trauma [12]. 3 patients had rapidly growing PEMs post trauma (6 weeks to 1 month between initial trauma and presenting symptoms). Only one case report had a reported duration of over 15 years between trauma and symptoms [12].

## 4 Conclusion

Although our case study does not prove trauma as a causative factor for her scalp PEM, the authors suggest that the patient's aetiology of her PEM could be from the entrapment of arachnoid islet cells secondary to her traumatic brain injury during infancy with a delayed adult onset in clinical presentation. This case study does demonstrate a classical presentation of PEM, that she was diagnosed via histopathological investigation, rather than clinical and radiographic findings. Interestingly in this case study, the patients' symptoms began 40 years post trauma. Other case studies of this rare tumour have correlated a shorter time period between the trauma and the diagnosis. We suggest that all patients should have both radiographic and histologic investigations of scalp masses that have history of head trauma, regardless of chronicity.

## Consent

Written informed consent was obtained from the patient for publication and any accompanying images. A copy of the written consent is available for review by the Editor-in-Chief of this journal on request.

## Ethical approval

Ethics approval was not necessary for this case study.

## Funding

No funding was received for this research.

## Author contribution

Elliot Crene – design of paper, writing paper, research, Claudia Cockburn – research data collection, literature review, editor, William Cockburn – concept of paper, primary surgeon data collector.

## Guarantor

Elliot James Douglas Crene.

## Declaration of competing interest

No conflicts of interest.

## References

[1] Greenberg M.S. (2019).

[2] Liu Y., Wang H., Shao H., Wang C. (2015). Primary extradural meningiomas in head: a report of 19 cases and review of literature. Int. J. Clin. Exp. Pathol..

[3] Kaye A.H. (2006).

[4] Özdemir ÖE, Özbek T, Tataroğlu C, Gürcan S, Örmeci AG. Extracranial meningioma: a case report. Meandros Medical and Dental Journal. 2018: 1;19(2):178–81.

[5] Shinichi S, Toranosuke I, Soichi I, Kazuo, M. Primary intracranial tumors among atomic bomb survivors and controls, Hiroshima and Nagasaki, 1961–75. 1980.

[6] Nygren C., Adami J., Ye W., Bellocoo R., Geijerstam. (2001). Primary brain tumors following traumatic brain injury – a population-based cohort study in Sweden. Cancer Causes Control.

[7] Rushing E.J., Bouffard J.-P., McCall S., Olsen C., Mena H., Sandberg G.D. (2009 May 20). Primary extracranial meningiomas: an analysis of 146 cases. Head Neck Pathol..

[8] Iaconetta G., Santella A., Friscia M., Abbate V., Califano L. (2012 Feb). Extracranial primary and secondary meningiomas. Int. J. Oral Maxillofac. Surg..

[9] Mattox A., Hughes B., Oleson J., Reardon D., McLendon R., Adamson C. (2010 Sep 7). Treatment recommendations for primary extradural meningiomas. Cancer.

[10] Prada F., Del Bene M., Moiraghi A., Casali C., Legnani F.G., Saladino A. (2015). From grey scale B-mode to elastosonography: multimodal ultrasound imaging in meningioma surgery—pictorial essay and literature review. Biomed. Res. Int..

[11] Liu N., Song S.-Y., Jiang J.-B., Wang T.-J., Yan C.-X. (2020 Feb 28). The prognostic role of Ki-67/MIB-1 in meningioma. Medicine.

[12] Yokoya S., Hisaoka S., Fujiwara G., Oka H., Hino A. (2022 Feb 18). Posttraumatic rapid growing extradural meningioma: a case report on the effectiveness of echosonography. Surg. Neurol. Int..

[14] Sohrabi C, Mathew G, Maria N, Kerwan A, Franchi T, Agha RA. The SCARE 2023 guideline: updating consensus Surgical CAse REport (SCARE) guidelines. Int J Surg Lond Engl. 2023;109(5):1136. 10.1097/JS9.0000000000000373 PMC10389401 37013953

[15] Agha R.A., Borrelli M.R., Farwana R., Koshy K., Fowler A., Orgill D.P., SCARE Group (2018). The PROCESS 2018 Statement: Updating Consensus Preferred Reporting Of CasE Series in Surgery (PROCESS) Guidelines. International Journal of Surgery.

[16] Yokoya S., Hisaoka S., Fujiwara G., Oka H., Hino A. (2022 Feb 18). Posttraumatic rapid growing extradural meningioma: a case report on the effectiveness of echosonography. Surg. Neurol. Int..

